# *Sphodromantis viridis* (Forskal, 1775): New for Portugal and new records of the rare and small mantids *Apteromantis aptera* (Fuente, 1894) and *Perlamantis allibertii* Guérin-Méneville, 1843 in the country (Mantodea: Mantidae and Amorphoscelidae)

**DOI:** 10.3897/BDJ.2.e1037

**Published:** 2014-01-08

**Authors:** Eduardo Marabuto, Ivo Rodrigues, Sérgio S Henriques

**Affiliations:** † CoBiG2 - Computational Biology and Population Genomics Group; CBA-FCUL, University of Lisboa, Lisboa, Portugal; ‡ Rua D. Afonso III, 22D, Beja, Portugal; § Terrestrial invertebrates Division, Department of Life Sciences, The Natural History Museum, London, United Kingdom

**Keywords:** New record, mantis, threatened species, distribution range, phenology, Iberian Peninsula

## Abstract

Several new records are presented on some of the least known mantis species in the Iberian Peninsula. From data collected in Portugal, their analysis has proven to represent an important advancement in the knowledge of this group of insects for the country and the Western Mediterranean area. Twenty new citations provide a better understanding on the distribution of the crepuscular species *Perlamantis allibertii*, the IUCN red listed *Apteromantis aptera* and the first Portuguese records of *Sphodromantis viridis*, extending their western limits of occurrence in Europe. The data thus gathered emphasizes the need to invest in biodiversity assessment for increased knowledge on species distribution and phenology but also for monitoring over time, essential to better ascertaining ecosystem services, the effects of climate change and habitat conservation.

## Introduction

Mantids are an easily recognised group of insects, mostly because of their characteristic raptorial front-legs, posture and worldwide distribution. However, beyond the basic concept of mantis, deeper distinction of many species within the Order Mantodea is still a matter confined to specialists and very keen observers. Many distinguishing characters rely on morphometric measurements and genitalia examination (e.g. Obertegger and Agabiti 2012), which represents an obvious impediment to the advancement in the knowledge of distribution and ecology of many species, especially the smallest ones and even in the well researched areas around the Mediterranean Sea. Here, in spite of a moderate species-richness of around 127 species, there is a clearly insufficient knowledge about many species, some of which recognisably threatened by ongoing processes such as desertification and habitat loss to urban areas or agriculture (Battiston et al. 2010).

35 mantis species have been cited for Europe, the Iberian Peninsula stands out as a hotspot for this group diversity with 14 species known for the area (Battiston et al. 2010, Ortuño and Martínez-Pérez 2011), although apparently only one, *Apteromantis aptera* (Fuente, 1884), is endemic. This species is the sole representative of the order Mantodea to be protected in the Habitats Directive and the Annex II of the Bern Convention (Peinado 1996), being also officially protected under Spanish legislation (Peinado and Mateos 1998).

While all 14 species cited for Iberia are known in Spain, only a rather depleted part of its diversity seems to reach its western limit in Portugal, where only eight species have been confirmed (Bolivar 1876; Bolivar 1897; Seabra 1942; Fernandes 1960; Grosso-Silva and Soares-Vieira 2004). Species whose presence in Portugal is yet to be validated include *Ameles decolor* (Charpentier, 1825), a central and eastern Mediterranean species which might have been mistaken for *Ameles nana* (Charpentier, 1825), already known from Portugal or with *Ameles picteti* (Saussure, 1869), currently known only from southern Spain and North Africa (Obertegger and Agabiti 2012). Further, the probable conspecificity of *Ameles spallanzania* (Rossi, 1972) and *Ameles africana* Bolivar, 1914 brings the number down to eight.

Because of the so called taxonomic impediment through the existence of not easily distinguished species and the lack of local taxonomists, knowledge about this group in Portugal has progressed slowly (Fernandes 1960, Grosso-Silva and Soares-Vieira 2004, Boieiro et al. 2007). The latest published study on Portuguese mantis by Boieiro et al. (2007) reports the second portuguese record of *Apteromantis aptera*, just three years after the publication first reporting it in the country and found the nocturnal species *Perlamantis allibertii* Guérin Méneville, 1843 after more than sixty years after its last record (Seabra 1937, Grosso-Silva and Soares-Vieira 2004) and thirty years after the previous 'official' mantis reference (Fernandes 1960). These latest studies have proven that field-work conducted on poorly prospected areas may provide new insights on the known distribution and ecology of threatened species and that thorough revisionary work is missing from Portugal accompanying the new advances on the study of Euro-Mediterranean Mantodea.

Hoping to fill the knowledge gap concerning Iberian Peninsula's mantis fauna and following field-work conducted over the last years, we report here a new species for Portugal, the African mantis, *Sphodromantis viridis* (Forskal, 1775) based on three independent sightings and seventeen new records on both small and probably overlooked species *Apteromantis aptera* (eight new records) and *Perlamantis allibertii* (9 new records). Altogether, we provide twenty new mantis records and summarise known information on the distribution, phenology and conservation of these three species in Portugal.

## Materials and methods

Insects were recorded mostly through two different survey types:

1. Active search, among scrub or under stones in suitable habitats, specimens were collected with appropriate insect-nets, examined and/or photographed *in situ* and then released. *Apteromantis aptera* was always located by this method.
2. Adults were attracted to 250 W Mercury Vapour bulbs over a white sheet suited for attracting moths or to porch lights. Individuals were then identified, examined and/or photographed before release. One specimen of *Sphodromantis viridis* (from Campo Maior, Portalegre) was collected and stored dry in the personal collection of the first author.

## Checklists

### Family Amorphoscelidae

Classification: Mantodea

#### Perlamantis allibertii

Guérin-Méneville, 1843

##### Materials

- **Type status:** Other material. **Occurrence:** occurrenceDetails: Seabra (1937, 1939); **Location:** country: Portugal; stateProvince: Alto Alentejo; county: Évora; locality: UTM: 29SNC96; verbatimLocality: Herdade da Mitra, Évora; **Event:** eventDate: 08-1938
- **Type status:** Other material. **Occurrence:** occurrenceDetails: Grosso-Silva & Soares-Vieira (2004); individualCount: 1; **Location:** country: Portugal; stateProvince: Beira Alta; county: Guarda; locality: UTM: 29TPF72; verbatimLocality: Castelo Rodrigo; **Event:** eventDate: 20-08-1997
- **Type status:** Other material. **Occurrence:** recordedBy: Sérgio Henriques; **Location:** country: Portugal; stateProvince: Trás-os-Montes; county: Bragança; municipality: Miranda do Douro; locality: UTM: 29TQF28; verbatimLocality: Vila Chã de Braciosa; locationRemarks: Natura 2000: PTZPE0038 / PTCON0022; verbatimLatitude: 41°25.20N; verbatimLongitude: 6°20.22W; verbatimCoordinateSystem: degrees decimal minutes; **Event:** samplingProtocol: ad hoc observation; eventDate: 12-09-2006; eventRemarks: under stone
- **Type status:** Other material. **Occurrence:** recordedBy: Valter Jacinto; **Location:** country: Portugal; stateProvince: Algarve; county: Faro; municipality: Castro Marim; locality: UTM: 29SPB22; verbatimLocality: Corte do Gago; verbatimLatitude: 37°17.39N; verbatimLongitude: 7°32.76W; verbatimCoordinateSystem: degrees decimal minutes; **Event:** samplingProtocol: porch light; eventDate: 26-06-2009
- **Type status:** Other material. **Occurrence:** recordedBy: Jorge Almeida, Pedro Pires, Paulo Rodrigues; individualCount: 1; **Location:** country: Portugal; stateProvince: Beira Baixa; county: Castelo Branco; municipality: Almaceda; locality: UTM: 29TPE12; verbatimLocality: Almaceda; verbatimLatitude: 40°00.65N; verbatimLongitude: 7°39.65W; verbatimCoordinateSystem: degrees decimal minutes; **Event:** samplingProtocol: light trap; eventDate: 21-08-2010
- **Type status:** Other material. **Occurrence:** recordedBy: Valter Jacinto; **Location:** country: Portugal; stateProvince: Algarve; county: Faro; municipality: Castro Marim; locality: UTM: 29SPB22; verbatimLocality: Corte do Gago; verbatimLatitude: 37°17.39N; verbatimLongitude: 7°32.76W; verbatimCoordinateSystem: degrees decimal minutes; **Event:** samplingProtocol: porch light; eventDate: 4-09-2010
- **Type status:** Other material. **Occurrence:** recordedBy: Eduardo Marabuto, Fernando Romão; individualCount: 5; **Location:** country: Portugal; stateProvince: Beira Alta; county: Guarda; municipality: Figueira Castelo Rodrigo; locality: UTM: 29TPF63; verbatimLocality: Faia Brava; locationRemarks: Natura 2000: PTZPE0039; verbatimLatitude: 40°57.17N; verbatimLongitude: 7°04.15W; verbatimCoordinateSystem: degrees decimal minutes; **Event:** samplingProtocol: light trap; eventDate: 11-09-2010
- **Type status:** Other material. **Occurrence:** recordedBy: Eduardo Marabuto, Samuel Infante; individualCount: 1; **Location:** country: Portugal; stateProvince: Beira Baixa; county: Castelo Branco; municipality: Monforte da Beira; locality: UTM: 29SPD49; verbatimLocality: Monte Barata; locationRemarks: Natura 2000: PTZPE0042; verbatimLatitude: 39°42.08N; verbatimLongitude: 7°18.90W; verbatimCoordinateSystem: degrees decimal minutes; **Event:** samplingProtocol: light trap; eventDate: 13-09-2010
- **Type status:** Other material. **Occurrence:** recordedBy: Eduardo Marabuto, Nélson Fonseca; individualCount: 3; **Location:** country: Portugal; stateProvince: Algarve; county: Faro; municipality: Silves; locality: UTM: 29SNB61; verbatimLocality: Algoz; verbatimLatitude: 37°09.66N; verbatimLongitude: 8°18.45W; verbatimCoordinateSystem: degrees decimal minutes; **Event:** samplingProtocol: light trap; eventDate: 6-08-2012
- **Type status:** Other material. **Occurrence:** recordedBy: Eduardo Marabuto, Nélson Fonseca; individualCount: 1; **Location:** country: Portugal; stateProvince: Algarve; county: Faro; municipality: Loulé; locality: UTM: 29SNB81; verbatimLocality: Fonte de Benémola; locationRemarks: Natura 2000: PTCON0049; verbatimLatitude: 37°12.58N; verbatimLongitude: 8°00.60W; verbatimCoordinateSystem: degrees decimal minutes; **Event:** samplingProtocol: light trap; eventDate: 7-08-2012
- **Type status:** Other material. **Occurrence:** recordedBy: Valter Jacinto; **Location:** country: Portugal; stateProvince: Algarve; county: Faro; municipality: Castro Marim; locality: UTM: 29SPB22; verbatimLocality: Corte do Gago; verbatimLatitude: 37°17.39N; verbatimLongitude: 7°32.76W; verbatimCoordinateSystem: degrees decimal minutes; **Event:** samplingProtocol: porch light; eventDate: 6-10-2012

##### Ecological interactions

###### Conservation status

Even though this species has a wide distribution, its elusive nocturnal habits (e.g. Bolívar 1914) reduce its chances to be seen. Therefore, its poorly known biology coupled with habitat loss resulting from conversion to intensive agriculture of scrubland and Mediterranean forest types has led Battiston et al. (2010) to attribute a "Potential risk" status to this species.

##### Distribution

This sole representative of the family Amorphoscelidae in Europe, this is an Atlanto-Mediterranean species generally distributed through the Iberian Peninsula, southern France and North Africa from Morocco to Tunisia (Battiston et al. 2010). In Spain, records are scattered although widespread (Correas 2009) and information on its biological requirements is scarce. Until now, solely known in Portugal from specimens collected in the thirties (Seabra 1937) and more recently, in 1997 (Grosso-Silva and Soares-Vieira 2004).

### Family Mantidae

Classification: Mantodea

#### Apteromantis aptera

(Fuente, 1894)

##### Materials

- **Type status:** Other material. **Occurrence:** occurrenceDetails: Grosso-Silva & Soares-Vieira (2004); **Location:** country: Portugal; stateProvince: Algarve; county: Faro; locality: UTM: 29SPB32; verbatimLocality: Malhão, Castro Marim; **Event:** eventDate: 7-03-2004
- **Type status:** Other material. **Occurrence:** occurrenceDetails: Boieiro et al. (2007); **Location:** country: Portugal; stateProvince: Baixo Alentejo; county: Beja; municipality: Castro Verde; locality: UTM: 29SNB97; verbatimLocality: , Herdade Belver, S. Marcos da Ataboeira; locationRemarks: Natura 2000: PTZPE0046; **Event:** eventDate: 2006-05-25/06-13
- **Type status:** Other material. **Occurrence:** recordedBy: Eduardo Marabuto, Ivo Rodrigues; individualCount: 2; **Location:** country: Portugal; stateProvince: Baixo Alentejo; county: Moura; municipality: Sobral da Adiça; locality: UTM: 29SPC50; verbatimLocality: Serra de Ficalho; locationRemarks: Natura 2000: PTCON0053; verbatimLatitude: 37°58.04N; verbatimLongitude: 7°16.63W; verbatimCoordinateSystem: degrees decimal minutes; **Event:** samplingProtocol: ad hoc observation; eventDate: 2-04-2008
- **Type status:** Other material. **Occurrence:** recordedBy: Sérgio Henriques; individualCount: 3; **Location:** country: Portugal; stateProvince: Alto Alentejo; county: Évora; municipality: Évora; locality: UTM: 29SNC86; verbatimLocality: Herdade da Mitra; verbatimLatitude: 38°31.62N; verbatimLongitude: 8°01.19W; verbatimCoordinateSystem: degrees decimal minutes; **Event:** samplingProtocol: ad hoc observation; eventDate: 24-10-2008
- **Type status:** Other material. **Occurrence:** recordedBy: Eduardo Marabuto; individualCount: 1; **Location:** country: Portugal; stateProvince: Baixo Alentejo; county: Beja; municipality: Beringel; locality: UTM: 29SNC81; verbatimLocality: 2km SW of Beringel; verbatimLatitude: 37°02.90N; verbatimLongitude: 7°59.93W; verbatimCoordinateSystem: degrees decimal minutes; **Event:** samplingProtocol: ad hoc observation; eventDate: 3-05-2009
- **Type status:** Other material. **Occurrence:** recordedBy: Sérgio Henriques; individualCount: 3; **Location:** country: Portugal; stateProvince: Algarve; county: Faro; municipality: Castro Marim; locality: UTM: 29SPB32; verbatimLocality: Azinhal; locationRemarks: Natura 2000: PTCON0036; verbatimLatitude: 37°17.19N; verbatimLongitude: 7°27.51W; verbatimCoordinateSystem: degrees decimal minutes; **Event:** samplingProtocol: ad hoc observation; eventDate: 15-03-2010
- **Type status:** Other material. **Occurrence:** recordedBy: Eduardo Marabuto, Fernando Romão; individualCount: 3; **Location:** country: Portugal; stateProvince: Baixo Alentejo; county: Beja; municipality: São Brissos; locality: UTM: 29SNC91; verbatimLocality: São Brissos quarry; verbatimLatitude: 37°04.85N; verbatimLongitude: 7°57.02W; verbatimCoordinateSystem: degrees decimal minutes; **Event:** samplingProtocol: ad hoc observation; eventDate: 10-04-2011
- **Type status:** Other material. **Occurrence:** recordedBy: Francisco Barros; individualCount: 1; sex: female; **Location:** country: Portugal; stateProvince: Alto Alentejo; county: Portalegre; municipality: Campo Maior; locality: UTM: 29SPD61; verbatimLocality: Nossa Senhora Expectação; locationRemarks: Natura 2000: PTCON0030; verbatimLatitude: 38°57.73N; verbatimLongitude: 7°04.62W; verbatimCoordinateSystem: degrees decimal minutes; **Event:** samplingProtocol: ad hoc observation; eventDate: 1-12-2011
- **Type status:** Other material. **Occurrence:** recordedBy: Sérgio Henriques; individualCount: >30; **Location:** country: Portugal; stateProvince: Baixo Alentejo; county: Beja; municipality: Castro Verde; locality: UTM: 29SNB96; verbatimLocality: Viseus; locationRemarks: Natura 2000: PTZPE0046; verbatimLatitude: 37°39.67N; verbatimLongitude: 7°57.40W; verbatimCoordinateSystem: degrees decimal minutes; **Event:** samplingProtocol: ad hoc observation; eventDate: 6-04-2012
- **Type status:** Other material. **Occurrence:** recordedBy: Francisco Barros; individualCount: 1; **Location:** country: Portugal; stateProvince: Alto Alentejo; county: Évora; municipality: Reguengos Monsaraz; locality: UTM: 29SPC25; verbatimLocality: Reguengos Monsaraz; verbatimLatitude: 38°25.90N; verbatimLongitude: 7°34.05W; verbatimCoordinateSystem: degrees decimal minutes; **Event:** samplingProtocol: ad hoc observation; eventDate: 28-05-2012

##### Ecological interactions

###### Conservation status

*Apteromantis aptera* is the only mantis species represented in the European Bern Convention (Annex II and IV) and Habitats Directive. Therefore it is the only species whose populations and habitats must be assessed periodically in the context of the Natura 2000 ecological network. Although this species is patchily distributed over the southern half of the Iberian Peninsula, it may be locally abundant (R. Obregón pers. com.). On overall, however, it is considered as "Seriously threatened" (Battiston et al. 2010) and is officially protected in Spain. Globally (although by the time it was assessed there were no Portuguese records), the IUCN still considers this species as "Near Threatened" (IUCN 1996), despite the expressed idea of a much needed revision.

##### Distribution

An Atlanto-Mediterranean species, this Iberian endemic mantis is generally limited to the southern parts of the Peninsula, where most records originate from Andalucia (Pascual Torres 2005, Obregón and López 2009) but is found up to the latitude of Madrid and Cuenca. The most up-to-date distribution map of the species in Spain is provided by Obregón and Gutiérrez (2013) who also provided new records for Huelva and Badajoz provinces, contiguous to the Portuguese territory and complement Pascual Torres (2012). Before, Pascual Torres (2011) projected the suitable distribution area for the species in Spain, with a high probability of occurrence near the border with Portugal to the south of the central mountain system. In Portugal, this species was found only twice, in March of 2004 (Grosso-Silva and Soares-Vieira 2004) and later by Boieiro et al. (2007) in the southeasternmost third of the country. In Morocco, *Apteromantis aptera* is replaced by vicariant sister species *Apteromantis bolivari* (Werner, 1931), where it is local and poorly known (Battiston et al. 2012).

#### Sphodromantis viridis

(Forskål, 1775)

##### Materials

- **Type status:** Other material. **Occurrence:** recordedBy: Eduardo Marabuto, Ivo Rodrigues; individualCount: 3; **Location:** country: Portugal; stateProvince: Alto Alentejo; county: Portalegre; municipality: Campo Maior; locality: UTM: 29SPD72; verbatimLocality: Castro; locationRemarks: Natura 2000: PTCON0030 / PTZPE0043; verbatimLatitude: 39°01.36N; verbatimLongitude: 6°58.17W; verbatimCoordinateSystem: degrees decimal minutes; **Event:** samplingProtocol: light trap; eventDate: 11-10-2008
- **Type status:** Other material. **Occurrence:** recordedBy: Ivo Rodrigues; individualCount: 1; **Location:** country: Portugal; stateProvince: Baixo Alentejo; county: Beja; municipality: Barrancos; locality: UTM: 29SPC72; verbatimLocality: Noudar; locationRemarks: Natura 2000: PTZPE0045 / PTCON0053; verbatimLatitude: 38°10.52N; verbatimLongitude: 7°02.38W; verbatimCoordinateSystem: degrees decimal minutes; **Event:** samplingProtocol: light trap; eventDate: 27-08-2011
- **Type status:** Other material. **Occurrence:** recordedBy: Ivo Rodrigues; individualCount: 1; sex: female; **Location:** country: Portugal; stateProvince: Baixo Alentejo; county: Beja; municipality: Moura; locality: UTM: 29SPC50; verbatimLocality: Serra de Ficalho; locationRemarks: Natura 2000: PTZPE0045 / PTCON0053; verbatimLatitude: 37°57.81N; verbatimLongitude: 7°16.75W; verbatimCoordinateSystem: degrees decimal minutes; **Event:** samplingProtocol: ad hoc observation; eventDate: 12-10-2013

##### Ecological interactions

###### Conservation status

According to (Battiston et al. 2010) this species is expected to be experiencing "favourable conditions" for its survival and expansion in the Mediterranean region, accounting for rising temperatures and changes in land use, with spreading of open semi-natural areas in detriment of strictly natural habitats.

##### Distribution

A chiefly Afrotropical species, widespread south of the Sahara but with more local populations to the north of this barrier around the Mediterranean (Battiston et al. 2010). For a long time, the only known European populations have been known from southern mainland Spain (chiefly Andalucia) (Bolivar 1897, Gangwere and Morales Agacino 1970). More recently it has been reported from the Balearic islands (Canyelles and Alomar 2006), presumably as the result of a human-assisted introduction.

## Analysis

**Figure 1.**
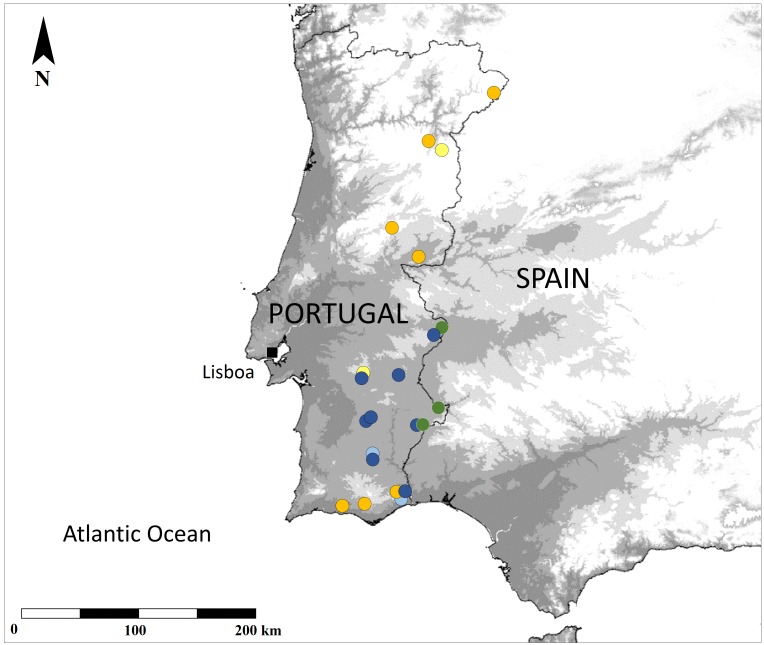
Occurence map of all records belonging to the three mantid species in Portugal. Light-coloured circles correspond to formerly published records while the dark are new records. Green circles: *Sphodromantis viridis*; Blue circles: *Apteromantis aptera*; Yellow circles: *Perlamantis allibertii*.

**Figure 3.**
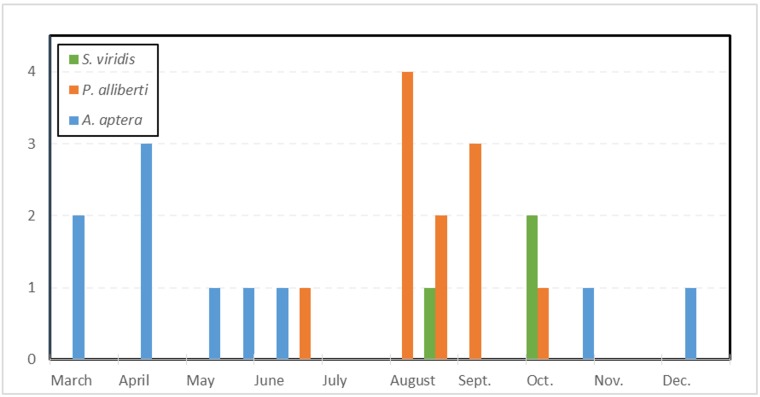
Recorded phenology for all Portuguese citations of *Perlamantis allibertii* (orange), *Apteromantis aptera* (blue) and *Sphodromantis viridis* (green). Diagram based on the data from Suppl. material 1.

Our field-work, based mostly on *ad-hoc* findings of Mantodea within the Portuguese territory over the last few years, found one distinctive, large species never before reported to the country and adds several relevant new records of two little known, smaller species. All the new records have been plotted against the previously known ones and are presented in Fig. 1, while pictures of live specimens of some of those records are presented on Fig. 2. We tentatively represent the perceived phenology of the three species according to all data currently available from Portuguese observations (Fig. 3).

Previous to this study, *Perlamantis allibertii* (Fig. 2d) was known from two very segregated localities, one in the interior of the country in Beira Alta and the other near Évora (Alto Alentejo). New records not only confirm its presence in the northern area as well as extend its distribution area by at least 60 km to the north near the Douro river, 155 km to the south and 33 km to the west in Algarve. In this last region, the three records attest its probable wider distribution. Moreover, we infer this species might be more widespread in the area in-between these extreme records as *Perlamantis allibertii* was found in the Tejo Internacional area (Monte Barata, Castelo Branco) and near Castelo Branco itself (Almaceda). Despite an isolated record from June, we can confidently ascribe this species's strongest activity period from August to the beginning of October (Fig. 3).

*Apteromantis aptera* (Fig. 2b), was previously known from two records within the same broad region of southeastern Portugal and the eight new sites extend the known range by at least 140 km to the north and 5 km to the west. Phenologically, the earliest record is from early March (Grosso-Silva and Soares-Vieira 2004) while the latest is from early December, although this species has been usually found during early April (Fig. 3). All specimens were located during day-searches and the inability to fly makes it improbable that this species can be recorded simultaneously with the other species surveyed, *i.e.* at lights during the night.

*Sphodromantis viridis* (Fig. 2a, c) is a complete novelty to the country and precise (published) distribution data in Spain is scarce. Because the three new records are so close to the international border with Spain, evidence remains to be put forward if the species is indeed breeding in Portuguese territory. Nevertheless, the late finding of a pregnant female (record c) makes it highly probable. Unlike *Apteromantis aptera* and in analogy to *Perlamantis allibertii*, all records of this species are concentrated during late Summer and especially early Autumn from August to October (Fig. 3).

The European Commission and EU member states have adopted and value certain areas which should be devoted to the survival and sensible management of biodiversity, termed Sites of Comunity Importance (SCI) under the Habitat's Directive, Birds Directive and Natura 2000 framework. In order to ease interpretation of data and to highlight the important finds reported in this study, we consider of relevance to summarise the presence of all three species within the boundaries of such SCI's (Table 1). Each of these important areas is coded differently if originally belonging to the Birds Directive (in Portugal starting with "PTZPE") or the Habitats Directive (in Portugal starting with the prefix "PTCON") and while some of them overlap over much of their area, this is not always the case.

## Discussion

The records here presented, notable finds such as a new species to the country and the considerable increase in the known distribution of two other species may not only be considered interesting but also remarkable. However, we think this only emphasizes the need for an increased effort in the knowledge of insect groups of paramount importance for ecological processes and conservation, where definitely both the largest mantis in Europe (*Sphodromantis viridis*) and the only officially protected species (*Apteromantis aptera*) must rank high.

### *Perlamantis allibertii*

Unlike most mantids in the area, *Perlamantis allibertii* is a typically nocturnal species which is best found at lights and thus requires a specific sampling protocol. Our several new records suggest it may be much more widespread and present than initially thought over Mediterranean-type habitats whenever their structure has been preserved. Altogether we extended the known distribution to the Algarve, 150 km to the south and to the west, around 30 km. However, some of the sites where it has been recorded are suffering pressures from urban or touristic infrastructures, especially in the Algarve. The case of the site in Algoz (Silves) is especially dramatic because this harbours an interesting endemic mediterranean vegetation community on a clay, acidic soil where one of the last remnant populations of the endangered quasi-endemic plant, *Plantago algarbiensis*, an European imperiled taxon considered Endangered by IUCN (Bilz 2011). This site is under serious threat because of its small dimensions and lies near the village of Algoz, is used to dump garbage and is right next to a quarry.

### *Sphodromantis viridis*

Besides being a new country record, the three Portuguese records of *Sphodromantis viridis* extend the distribution area of this species considerably to the west although precise reference data is scant. Nevertheless, Sevilla in Spain, not too far from the border is among the first known areas of the species back in the 19th century (Bolivar 1876). The fact that *Sphodromantis viridis* was only now recorded in Portugal despite being the largest mantid in Europe may probably be explained by one or the synergy between some factors: 1) the inland area of southern Portugal is on overall very poorly prospected and only recently the situation has changed slightly. Biodiversity assessments are underway but still do not fulfill the knowledge-gap; 2) possible confusion with the widespread and common Praying mantis, *Mantis religiosa* (Linnaeus, 1758) although upon close inspection, the presence of a white spot in the middle of the forewing and the absence of a yellow-centred dark spot in the fore-coxa give away for *Sphodromantis viridis* and 3) a possible on-going range expansion of this species from its core areas in Andalucia following climate and habitat conditions becoming more suitable in latest years. The relative contribution of each of these aspects can only be positively ascertained through careful examination of specimens collected in the past, among putative *Mantis religiosa* from the region, and newer records as well.

### *Apteromantis aptera*

The case of *Apteromantis aptera* may beslightly different. It is a distinctive wingless species (which immediately differentiates it from the often sympatric species in the genus *Ameles*) with pointed eyes and becomes well camouflaged in its natural habitats. Only reason 1 applies here because it is probably a poorly dispersive species very tied to its particular habitats, mostly semi-natural grassland and heliophilous scrubland. However, it is a species upon which there is an official concern and protection and most emerging records get to be published very soon after (Boieiro et al. 2007, Cano-Villegas and Zafra 2007, Grosso-Silva and Soares-Vieira 2004, Obregón and López 2009, Pascual Torres 2005, Pascual Torres 2011). We have found this species in some particularly rich areas concerning biodiversity. For instance, one record coming from the eastern foothills of Serra de Ficalho (Moura), a Natura 2000 priority site where its geology sets it apart from most other mountain ranges in southern Portugal. This area, although poorly surveyed harbours one of the few Portuguese populations of the Portuguese Dappled-white butterfly, *Euchloe tagis* (Hubner, 1804) (Marabuto 2008) and many orchid species. The second site near São Brissos (Beja) in spite of being small with less than 4 km^2^ is also of extreme importance. It is an abandoned semi-natural cork-oak woodland over a metamorphic carbonated rock patch very rich in Orchidaceae species (12 species so far), some of which are very rare in Portugal. Within this site there are many now abandoned marble quarries which provide refuges for fauna and flora and besides being next to the new São Brissos Airport, the area has been close to being completely leveled recently and if it would happen, most of these species would become locally extinct. The nearby site where *Apteromantis aptera* was found, near Beringel (Beja) is even smaller and equally of paramount importance for conservation. It is an abandoned traditional olive grove managed by Portuguese NGO QUERCUS-ANCN with a developed understorey harbouring the densest populations in Portugal of *Echium boissieri* (Boraginaceae). Furthermorem this is the type locality of the rare cicada *Euryphara contentei* Boulard, 1982, being the only area in Portugal where this species can be found (Sueur et al. 2004). Other sites include the important bird areas of Castro Verde and Caia, where relevant populations of great bustard (*Otis tarda*) and Lesser Kestrel (*Falco naumanii*) have their stongholds in the country.

### Conclusions

Taxonomic impediment as the generalised decrease of importance given to taxonomic aspects within biodiversity studies may be preventing many informed assessments and publishing of data (for instance, the recent find of a "Portuguese endemic" not easily identified cicada, *Tettigetalna mariae* Quartau & Boulard, 1995 in southern Spain is one such example (Simões et al. 2013)). The one thing in common these three mantis species share is the lack of knowledge regarding their biology and occurrence in Portugal and many of the problems associated with insect inventory and understanding of bionomics are expressed here. There is one large species likely to be able to disperse (and may be doing so with the help of climate amelioration) and moderately easy to detect; a potentially vagile species whose ecology (nocturnal) makes it difficult to be found in many biodiversity assessments and a third species that albeit legally protected and sought after is small, secretive, camouflages well among grasses and shrubs and is unlikely to undertake long distance dispersal.

While some records come from Sites of Community Importance (SCI) under the Natura 2000 framework which are subject to special management policies, several others do not. However, most come from biodiversity-rich places with no official or subject to little action in the field concerning conservation and even under immediate threat of local extinction. We hope our findings may help changing this situation and elevate the conservation profile of these species and their habitats.

## Supplementary Material

XML Treatment for Perlamantis allibertii

XML Treatment for Apteromantis aptera

XML Treatment for Sphodromantis viridis

## Figures and Tables

**Figure 2a.**
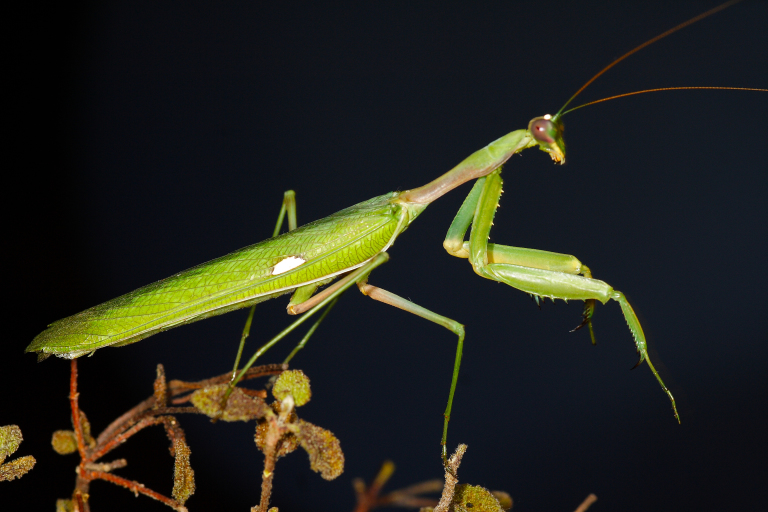
Male *Sphodromantis viridis* from near Campo Maior (Évora) – credit Eduardo Marabuto.

**Figure 2b.**
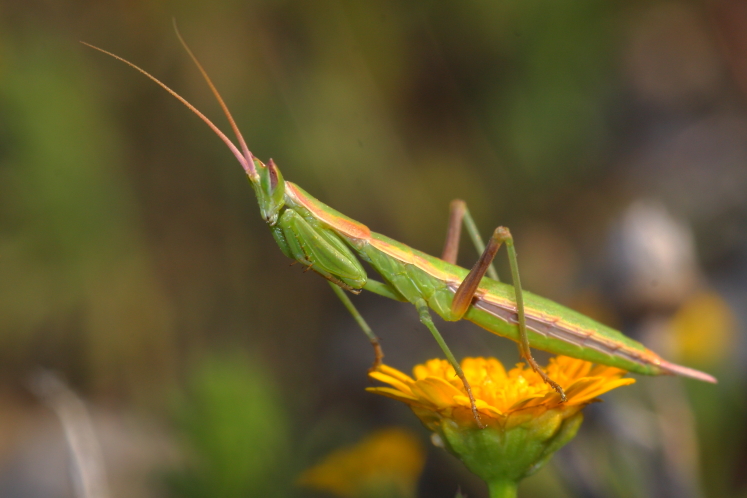
*Apteromantis aptera* from São Brissos (Beja) – credit Eduardo Marabuto.

**Figure 2c.**
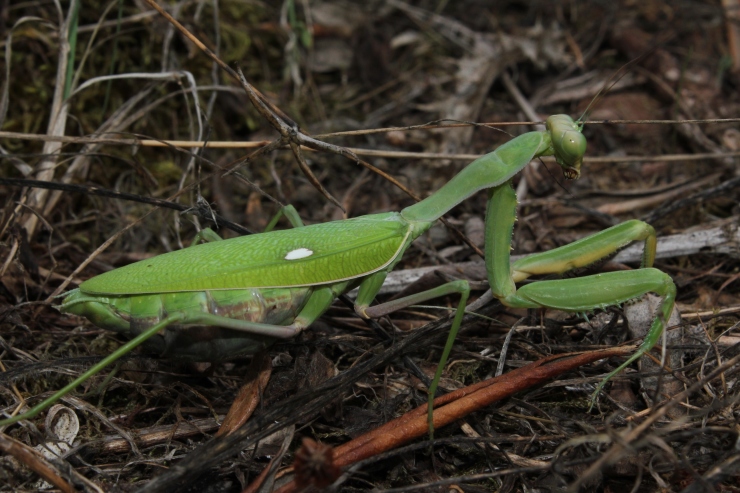
Female *Sphodromantis viridis* from Serra de Ficalho (Beja) – credit Ivo Rodrigues.

**Figure 2d.**
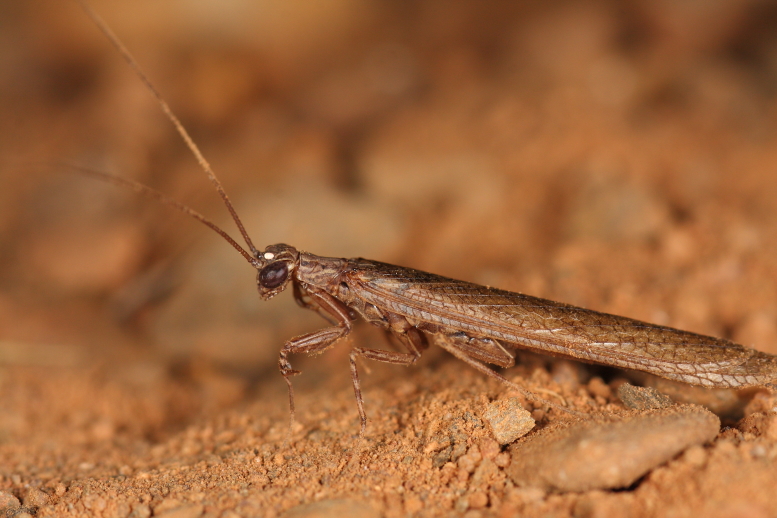
*Perlamantis allibertii* from Algoz (Faro) – credit Eduardo Marabuto.

**Table 1.** Presence of all known records of *Perlamantis allibertii*, *Apteromantis aptera* and *Sphodromantis viridis* within the Natura 2000 network of Portugal.

Species	Natura 2000 Sites of Community Importance
*Perlamantis allibertii*	PTZPE0038 (Douro Internacional e Vale do Águeda)
	PTZPE0039 (Vale do Côa)
	PTZPE0042 (Tejo Internacional, Erges e Pônsul)
	PTCON0022 (Douro Internacional)
	PTCON0049 (Barrocal)
*Apteromantis aptera*	PTZPE0045 (Mourão / Moura / Barrancos)
	PTZPE0046 (Castro Verde)
	PTCON0030 (Caia)
	PTCON0036 (Guadiana)
	PTCON0053 (Moura / Barrancos)
*Sphodromantis viridis*	PTZPE0043 (Campo Maior)
	PTZPE0045 (Mourão / Moura / Barrancos)
	PTCON0030 (Caia)
	PTCON0053 (Moura / Barrancos)

## Supplementary files

### Supplementary data to Figure 3

**Authors:** Eduardo Marabuto, Ivo Rodrigues, Sérgio Henriques **Data type:** phenological data **File:** oo_4991.pdf
