# Supplementary material for: Sphodromantis viridis (Forskal, 1775): New for Portugal and new records of the rare and small mantids Apteromantis aptera (Fuente, 1894) and Perlamantis allibertii Guérin-Méneville, 1843 in the country (Mantodea: Mantidae and Amorphoscelidae)
Source: Biodivers Data J. 2014 Jan 8;(2):e1037. doi: 10.3897/BDJ.2.e1037 (PMC3964699; doi:10.3897/BDJ.2.e1037)
Supplement: Supplementary file 4 — Authors: Eduardo Marabuto, Ivo Rodrigues, Sérgio Henriques Data type: phenological data File: oo_4991.pdf [file biodiversity_data_journal-2-e1037-s001.pdf]

**Species records**	**Week**
Sphodromantis viridis	
11/10/2008	Oct1
27/08/2011	Aug2
12/10/2013	Oct1
Perlamantis alliberti	
ago/37	Aug1
20/08/1997	Aug2
12/09/2006	Sept1
26/06/2009	June2
21/08/2010	Aug2
04/09/2010	Sept1
11/09/2010	Sept1
13/09/2010	Sept1
06/08/2012	Aug1
07/08/2012	Aug1
06/10/2012	Oct1
Apteromantis aptera	
07/03/2004	Marc1
01/06/2006	June1
02/04/2008	Apr1
24/10/2008	Oct2
03/05/2009	May1
15/03/2010	Marc1
10/04/2011	Apr1
01/12/2011	Dec1
06/04/2012	Apr1
28/05/2012	May2

Months

	S. viridis	*P. alliberti*	*A. aptera*
March			2
April			3
May			1
			1
June			1
		1	
July			
August		4	
	1	2	
Sept.		3	
Oct.	2	1	
			1
Nov.			
Dec.			1

Record data
week1 --> day 1-15
week2 --> day 16-31

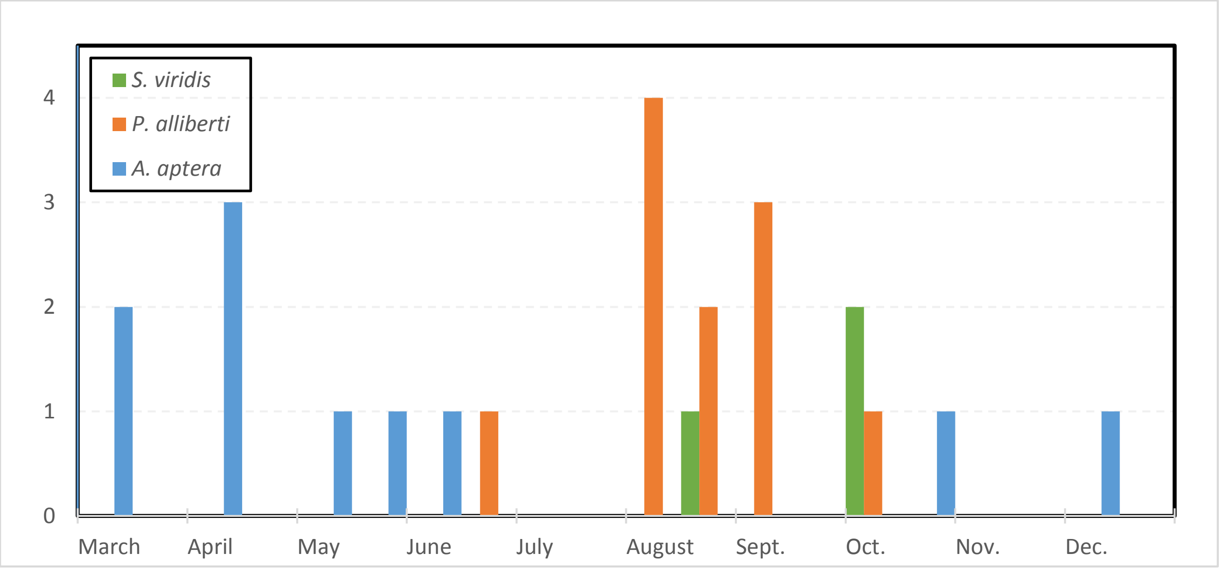
S. viridis
P. alliberti
A. aptera
0
1
2
3
4
March
April
May
June
July
August
Sept.
Oct.
Nov.
Dec.
